# Integrative approach on Pharyngodonidae (Nematoda: Oxyuroidea) parasitic in reptiles: Relationship among its genera, importance of their diagnostic features, and new data on *Parapharyngodon bainae*

**DOI:** 10.1371/journal.pone.0200494

**Published:** 2018-07-11

**Authors:** Felipe Bisaggio Pereira, José Luis Luque, Luiz Eduardo Roland Tavares

**Affiliations:** 1 Programa de Pós-Graduação em Biologia Animal, Instituto de Biociências, Universidade Federal de Mato Grosso do Sul, Campo Grande, Brasil; 2 Departamento de Parasitologia Animal, Instituto de Veterinária, Universidade Federal Rural do Rio de Janeiro, Seropédica, Brasil; Universidad Nacional Autonoma de Mexico, MEXICO

## Abstract

The first integrative approach using sequences of two genes (18S and 28S rRNA) plus morphological and life history traits, was explored in Pharyngodonidae nematodes parasitic in reptiles. Additionally, first genetic characterization of *Parapharyngodon bainae* and new data on its morphology are given. This approach evaluated the phylogenetic relationships among genera within Pharyngodonidae, as well as the importance of their diagnostic morphological features. Specimens of *P*. *bainae* were collected from faecal pellets of the lizard *Tropidurus torquatus* in the State of Minas Gerais, Brazil. Nematodes were fixed for scanning electron microscopy and molecular procedures. Morphological observations revealed the accurate structures of cephalic end, of cloacal region in males, of vulva and eggs. Phylogenetic reconstructions were based upon four datasets: aligned sequences of the 18S, of the 28S, of both concatenated genes and of combined morphological and molecular datasets. Bayesian inference and maximum likelihood were performed to infer the phylogenies of molecular datasets and maximum parsimony to infer that of all-combined data. Pharyngodonid parasites of reptiles seem to configure two general monophyletic lineages, as previously assertions. Results also showed the monophyly of *Spauligodon*, *Skrjabinodon* and *Parapharyngodon*, as well as the clear separation between the latter and *Thelandros*. Combination of datasets improved nodal supports. Analysis of the all-combined datasets revealed the importance of vulval position and egg morphology as phylogenetic informative traits. However, characters of male caudal morphology appear as are highly homoplastic, and seem to be product of convergent evolution or multiple losses of ancestral traits. The closely-related *Thelandros* and *Parapharyngodon* are kept valid and their diagnosis should be based upon the position of the operculum in eggs (terminal or subterminal, respectively). Some inconsistencies in the scarce molecular and morphological databases were noted. Thus, new genetic data is required for further conclusions and current database must be evaluated with attention.

## Introduction

Pharyngodonidae is a diverse family of oxiuroid nematodes parasitic in all classes of vertebrates, except Aves [1]. Despite 24 genera have been allocated in Pharyngodonidae [1–3], the morphological aspects of several taxa remain poorly studied, which results in unclear diagnosis. A very illustrative example is regarding *Parapharyngodon* and *Thelandros*, closely-related genera with similar morphological aspects, considered synonyms by some authors and independent by others (e.g., [1,4–7]). Therefore, their boundaries are ill-defined and complicated [8].

Genetic database on pharyngodonids is restricted to the parasites of lizards of few species belonging to few genera (e.g.[9–12]). This fragmented database along with incomplete morphological knowledge on a substantial number of species, complicate the understanding of phylogenetic patterns among these parasites. Moreover, the generic diagnosis and validity of some taxa remain poorly resolved. Therefore, integrative approaches using new genetic and morphological data on pharyngodonid nematodes may represent important tools to clarify such issues.

During a genetic and morphological study pertaining to *Parapharyngodon bainae*, a parasite of the lizard *Tropidurus torquatus* from Brazil, we decided to take advantage of the current genetic database and perform the first integrative approach on Pharyngodonidae parasitic in reptiles. The objectives were to evaluate the phylogenetic relationships among the genera, discuss their validity as well as the importance of the most representative diagnostic traits, with emphasis on *Parapharyngodon* and *Thelandros*. Additionally, new morphological and genetic data for *P*. *bainae* is provided, including the first observation of the species using scanning electron microscopy (SEM).

## Materials and methods

### Collecting, processing and morphological examination

During 2013, several nematodes were collected alive from fresh faecal pellets of the lizard *T*. *torquatus*, in a rocky outcrop area from the district of Toledos, Municipality of Juiz the Fora, State of Minas Gerais, Brazil (21°48'S, 43°35'W; altitude 697m). Permission for land use was guaranteed by the Instituto Brasileiro do Meio Ambiente e dos Recursos Naturais (acronym IBAMA; Process 0.2015.010660 / 05–88 license no. 261 / 05-NUFAS / MG), since collection site was on a federal area. Lizards were caught actively by loop traps and kept individually in adequate plastic boxes, placed under shade, until defecation, being released shortly thereafter; animals were kept trapped for no more than 10 minutes. All procedures involving animal manipulation were permitted by the IBAMA (Process 0.2015.010660 / 05–88 license no. 261 / 05-NUFAS / MG) and were in strict accordance with the recommendations of the Colégio Brasileiro de Experimentação Animal (acronym COBEA). The protocol was approved by the Committee on Ethics of Animal Experiments of the Universidade Federal de Juiz de Fora (Protocol Number: 010/2005-CEA). Parasites were removed from faecal pellets, washed in saline (0.9% NaCl), fixed in hot 4% formalin and preserved in 70% ethanol. Before fixation in formalin, the middle body parts of five specimens were excised and fixed in molecular grade 96–99% ethanol for genetic studies. Nematodes were identified based on [13]; the systematic classification of higher taxa follows [1], except that *Thelandros* and *Parapharyngodon* were not considered synonyms as suggested in recent publications [14–16]. Four males and four females, used for SEM, were dehydrated through a graded ethanol series, dried in hexamethyl disilazane, coated with gold and examined in a JEOL JSM-740 1F, at an accelerating voltage of 4 kV. Voucher specimens were deposited in the Coleção Helmintológica do Instituto Oswaldo Cruz (accession no. CHIOC38373).

### DNA isolation, PCR and sequencing

Genomic DNA was isolated from tissue samples using DNeasy Blood and Tissue Kit (QUIAGEN, Hilden, Germany), following manufacturer’s instructions. The SSU rRNA gene (18S) was amplified in PCR reactions (25μl) consisted of 2.5 μl of 10X PCR buffer minus Mg, 1.0 μl of MgCl_2_ (50mM), 2 μl of dNTP’s (2.5mM), 0.25 μl of each oligonucleotide primer (10μM), 0.2 μl of Platinum Taq DNA polymerase (5 U/μl) (Invitrogen, Carlsbad, California), 0.25 μl of BSA, 16.5 μl of H_2_O and 2.0 μl of genomic DNA, using the PCR conditions and the primers Philonema F + PhilPCRr described in [17]. The LSU rRNA gene (28S) was amplified in PCR reactions (25μl) consisted of 2.5 μl of 10X PCR buffer minus Mg, 1.5 μl of MgCl_2_ (50mM), 2 μl of dNTP’s (2.5mM), 0.25 μl of each oligonucleotide primer (10μM), 0.2 μl of Platinum Taq DNA polymerase (5 U/μl) (Invitrogen, Carlsbad, California), 0.25 μl of BSA, 16.0 μl of H_2_O and 2.0 μl of genomic DNA, using the primers D2A + D3B of [18]. The cycling parameters for amplification of the 28S rDNA were as follows: denaturation at 94°C for 5 min, followed by 35 cycles of 94°C for 15s, annealing at 50°C for 20s and elongation at 72°C from 30s, followed by a final post-amplification extension at 72°C for 5 min. PCR products were purified through an enzymatic treatment with exonuclease I and shrimp alkaline phosphatise [19], and Sanger sequenced in GATC Biotech (Konstanz, Germany) using the PCR primers and two additional internal primers (WF760 and WR800 see [17]) in the case of 18S rDNA. Contiguous sequences were assembled in Geneious (Geneious ver. 9 created by Biomatters, available from http://www.geneious.com/) and deposited in GenBank database under accession numbers MF102080 / MF102081.

### Phylogenetic analyses of molecular data

Sequences used in the present study are listed in Table 1 and were chosen according the following criteria: sequence length (> 750 bp for 18S covering most of the 5' half of the gene, and > 750 bp for 28S), generated from species allocated in Pharyngodonidae, availability in GenBank database and congruence of genetic region according to alignment algorithms. Because of the numerous similar sequences available for the same gene from a same species, we decided to use sequences from just one isolated in the case of *Skrjabinodon* spp., *Spauligodon* spp. and *Parapharyngodon cubensis*. The sequences GU992864 and JN020352, supposedly from *Parapharyngodon sceleratus*, were excluded because they do not match with other sequences of pharyngodonids, blast search showed high similarity with ascaridoid nematodes, these sequences were not published in scientific papers and the isolation source is not linked to any morphological identification. Phylogenetic analyses were based upon four different datasets: (i) alignment of the 18S sequences, (ii) alignment of the 28S sequences, (iii) concatenated alignment of both genes and (iv) morphological and life history data combined with that from molecular alignments of the concatenated genes.

**Table 1:** Samples whose sequences were retrieved from GenBank and were used in phylogenetic analysis, associated with host, locality, gene and accession number.

Sample	Host	Locality	Gene	Accession Number	Reference
*Ozolaimus linstowi*	*Iguana iguana*	Mexico	18S; 28S	KJ632671; KJ632667	[20]
*Parapharyngodon cubensis*	*Anolis pulchellus*	Puerto Rico	18S	KF029168	[21]
*Parapharyngodon echinatus* 1	*Gallotia atlantica mahoratae*	Spain	18S; 28S	JF829224; JF829241	[22]
*Parapharyngodon echinatus* 2	*Tarentola pervicarinata*	Senegal	18S	AM943009	[23]
*Parapharyngodon sceleratus* 1^a^	*Hemidactylus brooki*	India	18S	KC335146	Unpublished
*Parapharyngodon sceleratus* 2^a^	*Hemidactylus brooki*	India	18S	KP338604	Unpublished
*Skrjabinodon poicilandri*	*Woodworthia maculata*	New Zealand	18S; 28S	KX550036; KX550055	[9]
*Spauligodon anolis*	*Anolis cristatellus*	Puerto Rico	18S	KF029004	[21]
*Spauligodon atlanticus*	*Gallotia atlantica mahoratae*	Spain	18S; 28S	KJ778075; KJ778099	[10]
*Spauligodon auziensis*	*Tarentola mauritanica*	Morocco	18S; 28S	JF829225; JF829242	[22]
*Spauligodon carbonelli*	*Podarcis hispanica*	Spain	18S; 28S	JF829229; JF829248	[22]
*Spauligodon lacertae*	*Lacerta media*	Armenia	18S; 28S	JF829236; JF829254	[22]
*Spauligodon nicolauensis*	*Tarentola bocagei*	Cape Verde	18S; 28S	JF829226; JF829243	[22]
*Spauligodon saxicolae*	*Darevskia bendimahiensis*	Turkey	18S; 28S	KJ778084; KJ778093	[10]
*Thelandros tinerfensis*	*Tarentola gomerensis*	Spain	18S; 28S	KX778073; KX778089	[10]
*Trypanoxiuris pigrae*	*Alouatta pigra*	Mexico	18S; 28S	KU285458; KU285469	[24]
*Skrjabinodon* sp.	*Dactylocnemis pacificus*	New Zealand	18S; 28S	KX550038; 550056	[9]
*Spauligodon* sp.	*Oligosoma polychroma*	New Zealand	18S; 28S	KX550022; KX550043	[9]
*Thelandros* sp.	Not specified	Not specified	28S	KF771647	Unpublished

^a^Wrongly nominated as *Thelandros sceleratus* in the GenBank.

The 18S and 28S datasets were aligned separately using T-Cofee [25,26], then subjected to the transitive consistency score [27] for estimation of the alignment accuracy and trim ambiguously aligned positions. Trees were generated from all four datasest. Gene alignments were subjected to maximum likelihood (ML) and Bayesian inference (BI) using PHYML [28] and MrBayes [29], respectively, under the following models of evolution TIM2 + I + G for 18S, TPM3uf + G for 28S and GTR + I + G for the concatenated datasets, chosen according to the Akaik Information Criterion using jModel Test 2 [28,30]. For ML analysis bootstrap resampling was performed with 1,000 replications. Bayesian posterior probability values from BI, were determined after running the Markov chain Monte Carlo (2 runs 4 chains) for 4 × 10^6^ generations, with sampling frequency every 4 × 10^3^ generation and discarding the initial 1/4 of sampled trees (1 × 10^6^) as burn-in.

### Morphological and life history data coding, character mapping and integrated analysis with molecular data

This analysis included only samples identified to specific level, and their morphological data was gathered directly from their respective taxonomic descriptions (see Table 2). Characters and states for parasite morphological and life history data matrix were chosen and coded according to what [1,2] considered to have systematic/phylogenetic relevance; related literature regarding the biology of the respective hosts was evaluated for some life history traits; all these information are detailed in Table 2. The characters and states were generated according to the following criteria: main features that diagnose the genera within Pharyngodonidae (see [1]) and highlighted traits that have been used for separate *Thelandros* from *Parapharyngodon* (see [31]), since they are the most problematical taxa in the family. Data of the morphological-life history traits matrix combined with molecular datasets of concatenated genes (18S + 28S) were generated using Mesquite [32]. Using PAUP (version 4.0a152) [33], all-combined data matrix was partitioned in three categories (morphological + 18S + 28S) and the incongruence length difference test (partition homogeneity test) was performed to evaluate if the combinations of these partitions would increase phylogenetic accuracy [34]. A tree from the all-combined datasets was inferred using maximum parsimony (MP) analysis with 2,000 bootstrap replications, and examination of the most parsimonious distribution of character states on this tree were performed in PAUP and Mesquite.

**Table 2:** Life history and morphological characters and states^a^ associated with the taxa used in the all-data integrated analysis.

Sample	Host Class	Host diet	Lateral alae	Caudal alae	Genital cone	Pedunculate papillae	Tail filament	Vulval position	Egg structure	Egg shape	References
*Ozolaimus linstowi*	Reptilia	herbivorous	absent	present	present	present	absent	posterior	without operculum	oblate spheroid	[20]
*Parapharyngodon bainae*	Reptilia	omnivorous	present in male	absent	absent	absent	developed	median	single subterminal operculum	oblate spheroid	[13]; present study
*Parapharyngodon echinatus* 1	Reptilia	omnivorous	present in male	absent	present	present	developed	median	single subterminal operculum	oblate spheroid	[23]
*Skrjabinodon poicilandri*	Reptilia	omnivorous	present in both sexes	absent	absent	absent	developed	anterior	without operculum	spindle-shaped	[35]
*Spauligodon atlanticus*	Reptilia	omnivorous	present in both sexes	present	present	absent	developed	anterior	2 polar opercula	spindle-shaped	[12]
*Spauligodon auziensis*	Reptilia	omnivorous	present in both sexes	present	present	present	developed	anterior	2 polar opercula	spindle-shaped	[36]; [37]
*Spauligodon carbonelli*	Reptilia	omnivorous	present in both sexes	present	present	present	developed	anterior	2 polar opercula	spindle-shaped	[38]
*Spauligodon lacertae*	Reptilia	omnivorous	present in both sexes	present	present	present	developed	anterior	2 polar opercula	spindle-shaped	[39]
*Spauligodon nicolauensis*	Reptilia	carnivorous	present in male	present	present	present	developed	anterior	single terminal operculum	spindle-shaped	[11]
*Spauligodon saxicolae*	Reptilia	omnivorous	present in both sexes	present	present	present	developed	anterior	2 polar opercula	spindle-shaped	[39]
*Thelandros tinerfensis*	Reptilia	carnivorous	present in male	present	present	present	developed	median	single terminal operculum	oblate spheroid	[40]
*Trypanoxiuris pigrae*^b^	Mammalia	herbivorous	present in both sexes	present	absent	present	reduced	anterior	without operculum	oblate spheroid	[24,41]

^a^Characters and states were selected based on [1,2].

^b^Outgroup.

## Results

**Fig 1:**
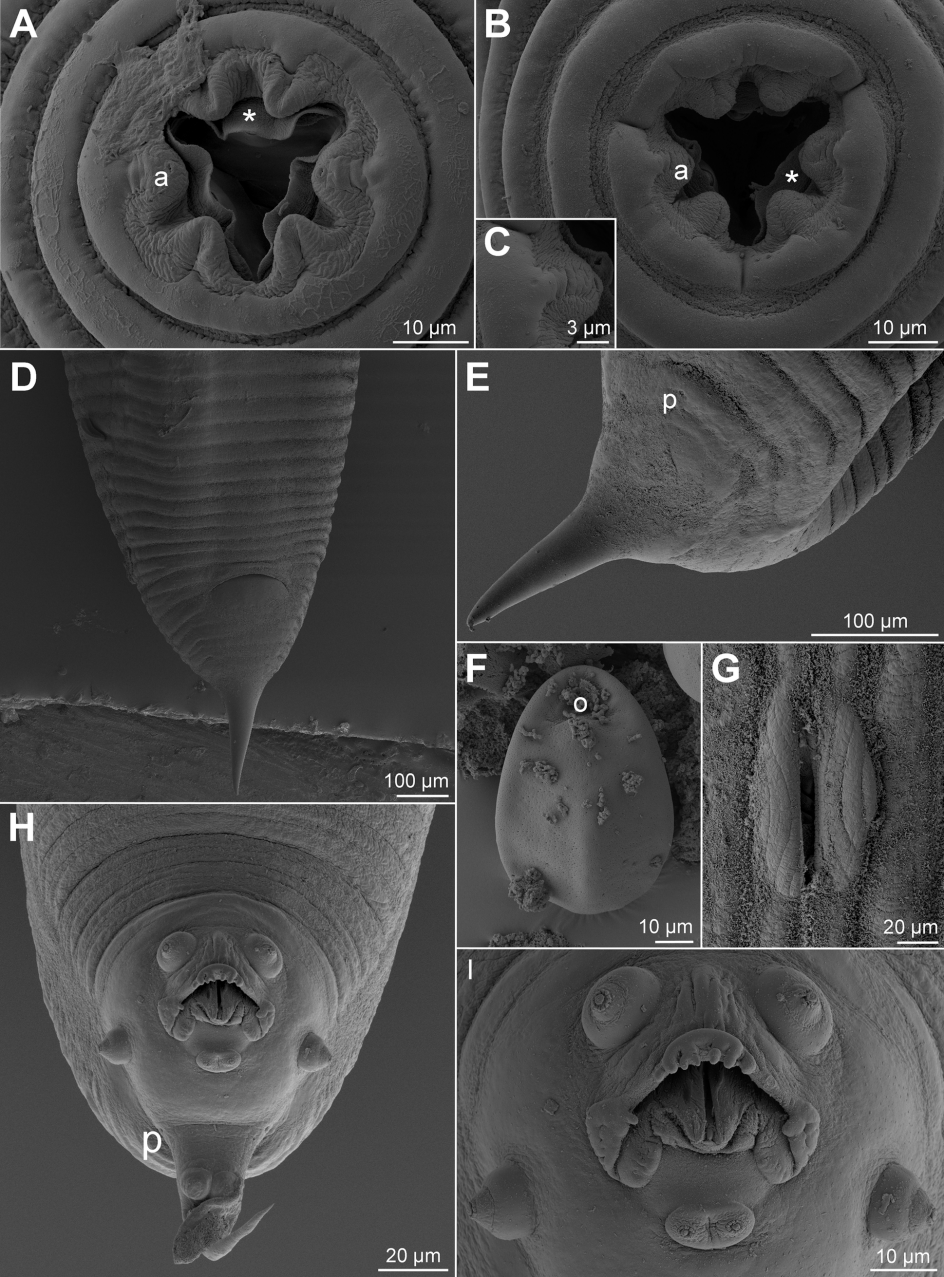
Scanning electron micrographs of *Parapharyngodon bainae* collected from faecal pellets of *Tropidurus torquatus*. A, B, Cephalic end of female and male, respectively, apical views (asterisks indicate lamellar projections). C, Detail of amphidial pore in lateral labium. D, E, Tail of female, ventral and lateral views, respectively. F, Egg. G, Vulva, apical view. H, I, Tail and cloacal region of male, respectively, ventral views (arrowhead indicates spicule tip). Abbreviations: a, amphid; o, operculum; p, phasmid.

Morphological and biometric results (S1 Table) indicated that the newly collected nematodes belong to *P*. *bainae*. SEM observations revealed the following features: no sexual dimorphism in the morphology of the cephalic end, six lips surrounding the oral opening, of which two subdorsal, two subventral and two lateral (Fig 1A and 1B). Subdorsal and subventral labia without papillae (Fig 1A and 1B); lateral labia with minute amphidial pores (Fig 1C). Lamellar structures just below the labia, projecting to the center of oral cavity (Fig 1A and 1B). Female with stout and long terminal spike in tail, phasmidial pores about 250 μm from tail tip (Figs D, E); ellipsoid eggs with subterminal operculum (Fig 1F); vulval labia protruded (Fig 1G). Male with three pairs of caudal papillae, of which first pair precloacal and subventral, second pair lateral and slightly postcloacal, third pair in tail filament, plus one ventral postcloacal double papillae (Fig 1H and 1I). Minute phasmidial pores laterally located in the basis of the caudal filament (Fig 1H). Anterior cloacal lip with echinate median edge and two lateral swellings; potcloacal lip well-developed, forming a sheath-like structure that surrounds cloacal opening and distal end of spicule (Fig 1I).

Partial sequences of the 18S (1464 bp) and 28S (783 bp) rRNA genes of *P*. *bainae* were obtained. Sequences were identical among the five samples taken for molecular study, therefore only one representative was used in the phylogenetic reconstructions.

**Fig 2:**
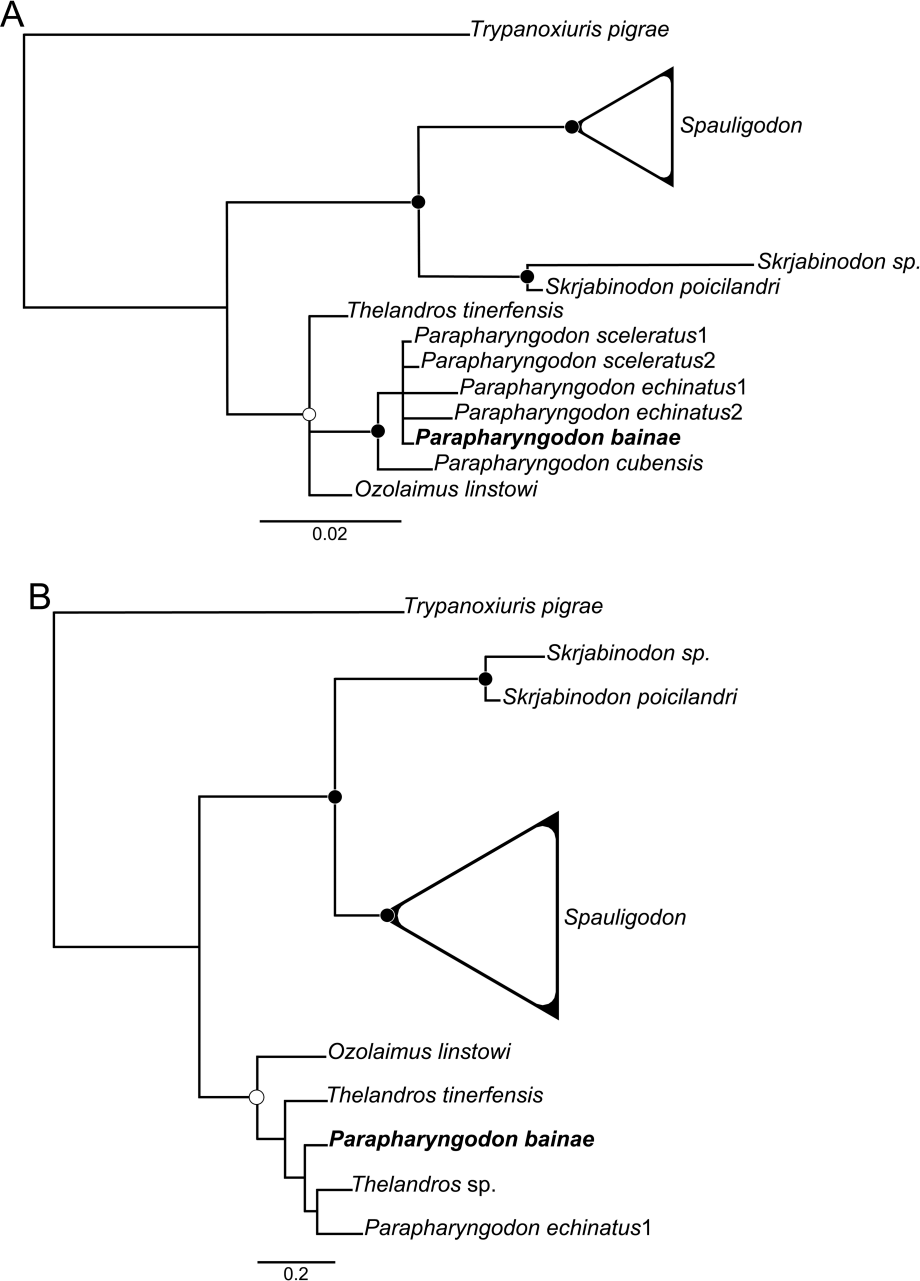
**Phylogenetic trees generated using Bayesian inference of the sequences of 18S (A) and 28S (B) rDNA alignments from pharyngodonid nematodes parasitic in reptiles**. Full and empty circles indicate nodal support > 0.96 and > 0.80, respectively, for Bayesian posterior probability (4 × 10^6^ generations, sampling frequency = 4 × 10^3^, burn-in = 1 × 10^6^) and > 96 and > 80, respectively, for maximum likelihood bootstrap (1,000 replications). Branches formed by *Spauligodon* spp. were collapsed. Specimen in bold is from the present study.

**Fig 3:**
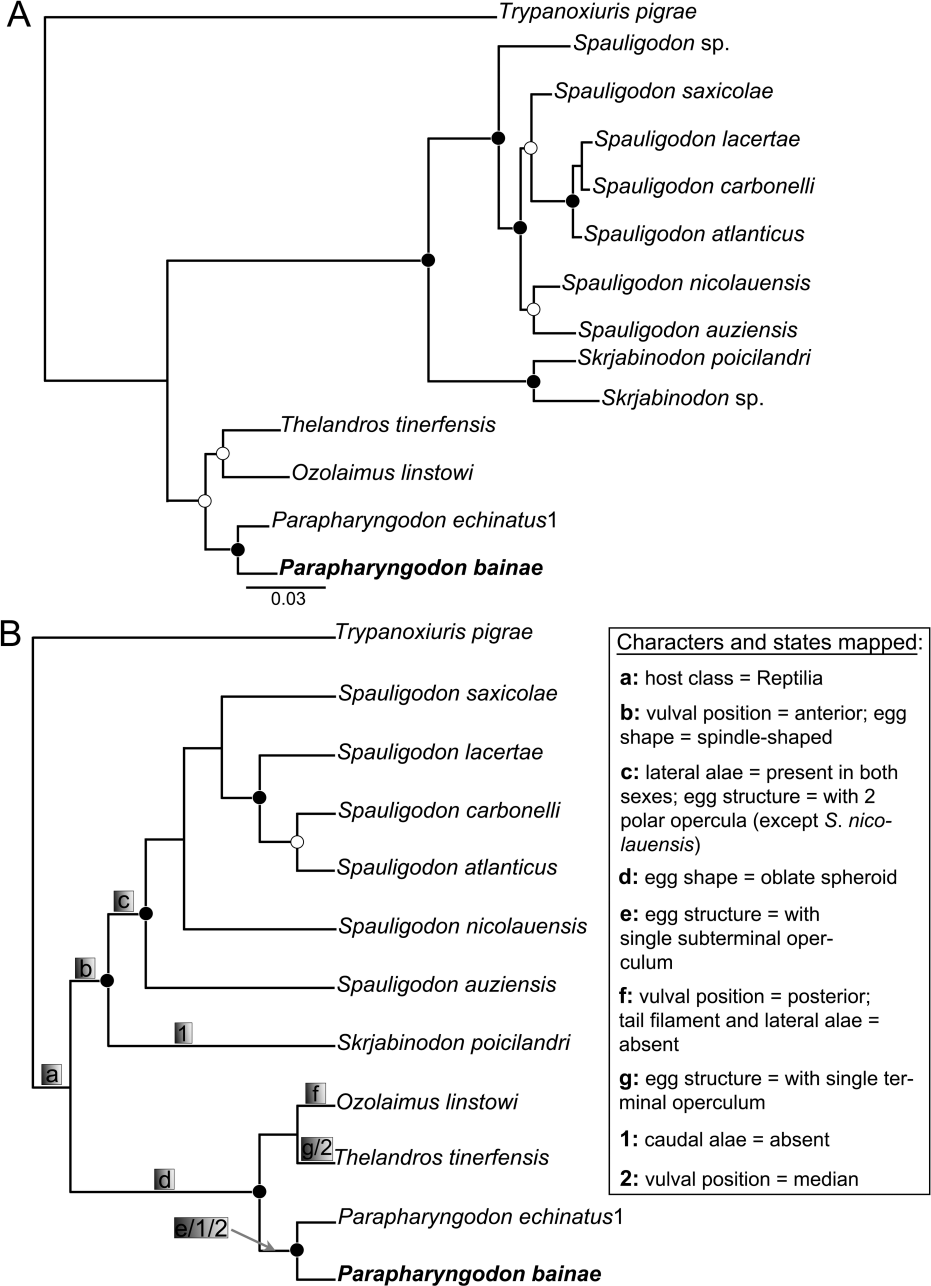
Phylogenetic relationships among pharyngodonid nematodes parasitic in reptiles. (A) Tree generated using Bayesian inference of the concatenated sequences of 18S and 28S rDNA; full and empty circles indicate nodal support > 0.96 and > 0.80, respectively, for Bayesian posterior probability (4 × 10^6^ generations, sampling frequency = 4 × 10^3^, burn-in = 1 × 10^6^) and > 96 and > 80, respectively, for maximum likelihood bootstrap (1000 replications). (B) Tree generated using maximum parsimony of the combined data from morphological-life history and molecular datasets (2423 characters, 274 parsimony informative, CI 0.786); full and empty circles indicate bootstrap support > 96 and > 80, respectively (2,000 replications); life history and morphological traits are mapped and labelled in details. Specimen in bold is from the present study.

The topology of the phylogenetic trees generated using ML and BI were very similar (for ML trees see S2 Fig, S3 Fig and S4 Fig). In the cladograms from molecular data the genera *Parapharyngodon*, *Spauligodon* and *Skrjabinodon*, represented by more than one species, were monophyletic (Figs 2A, 2B and 3A). *Thelandros* formed an independent lineage from *Parapharyngodon* (Figs 2A, 3A and 3B), except in the tree inferred from the 28S dataset (Fig 2B). In the phylogenetic reconstructions using the 18S and 28S separately, the generic assemblages formed by *Sapuligodon* spp. were collapsed (Fig 2A and 2B), because the objective was to evaluate only the intergeneric relationships. Phylogenetic reconstruction and nodal supports improved considerably when molecular datasets from the two genes were concatenated (Fig 3A).

The partition homogeneity test indicated that combining datasets increased the phylogenetic accuracy (p = 0.16). The integrated analysis using morphological and molecular datasets (2423 characters, 274 parsimony informative characters) produced one most parsimonious tree (steps 1396, consistency index [CI] 0.786) (Fig 3B). Morphological and life history traits were explored in the MP tree (Fig 3B). The cladogram generated from the morphological-life history traits matrix is shown in S1 Fig. According to the CI values, evolution of most characters was explained by the minimum number of required changes, and few were highly homoplastic (Table 3).

**Table 3:** Characters (morphological and life history) that were mapped in the maximum parsimony tree associated with states, no. of steps and consistency index (CI).

Character	States^a^	No. of steps	CI
Host Class	2	1	1.00
Host diet	3	4	0.50
Lateral alae	3	3	0.67
Caudal alae	2	2	0.50
Genital cone	2	3	0.33
Pedunculate papillae	2	3	0.33
Tail filament	3	2	1.00
Vulval position	3	2	1.00
Egg structure	4	4	0.75
Egg shape	2	2	1.00

^a^States were coded according to what is shown in Table 2.

## Discussion

The newly collected specimens were morphologically and biometrically identical to those described by [13]; furthermore, samples were recovered from the type host (*T*. *torquatus*) in the type locality (Toledos, Minas Gerais, Brazil), indicating that the present material belong to *P*. *bainae*. This first observation of *P*. *bainae* using SEM, revealed the following traits inaccurately described or overlooked in the original description: labial papillae absent, presence of lamellar projections bellow the labia, location of phasmids in males and females, protruded vuval labia in females, structure of cloacal lips and the postcloacal double papilla in males. These findings are important, since *Parapharyngodon* retains high number of poorly described species and most of them were not observed using SEM [8,16].

The present analysis using genetic sequences confirmed the validity of *P*. *bainae*, in which the species formed an independent lineage from the other congeners as well as from other pharyngodonids (Figs 2B, 3A and 3B). However, in the phylogenetic reconstruction using the 18S (Fig 2A) the position of *P*. *bainae* was unresolved within lineages of *Parapharyngodon*. Some authors consider the 18S alone poorly informative for species scrutiny rather than the D2-D3 region of the 28S rRNA [42,43], regarding nematodes parasites of vertebrates. In this sense, the most adequate is to combine more than one dataset, as shown in the present work and previous related approaches (e.g. [9,10,22]).

Even though some studies deal with numerous sequences of the same gene from a single species [9–11,21,22], few species belonging to few genera of Pharyngodonidae have been genetically characterized (i.e. 5 genera out of 24) [9,10,20–23]. Furthermore, some available sequences (mainly those that have been not published in previous papers) are seemingly incorrect, e.g., the 28S sequences of *P*. s*celeratus* (GU992864, JN020352), wrongly nominated as *T*. *sceleratus*, appeared closer to Ascaridoidea than to Pharyndogonidae in a BLAST search. Moreover, the referred sequences were generated from isolates collected in India, whereas *P*. *sceleratus* is a parasite found in lizards from the Neotropics (see [16]).

The weakness of some traits used on generic diagnosis together with fragmented database, complicate the boundaries between some taxa of Pharyngodonidae. The most expressive example is the lack of consensus regarding the validity of *Thelandros* and *Parapharyngodon* [1,4–6]. Based on the phylogenetic reconstructions, *Parapharyngodon* formed an independent lineage from *Thelandros* (Figs 2A, 3A and 3B), except on the tree generated from the alignment of 28S sequences (Fig 2B). In this case, *Thelenadros* sp. clustered between representatives of *Parapharyngodon*. Most likely, this isolate was misallocated in *Thelandros*, since both genera have very similar morphology. Furthermore, there is no information on the morphology of this supposed *Thelendros* sp., data have not been published and no voucher (or hologenophore) has been deposited in parasitological collections.

Even though the tree generated from the 28S dataset has revealed poor resolution among *Thelandros* and *Parapharyngodon* (Fig 2B), trees form other datasets strongly supported the monophyly of *Parapharyngodon*, as well as the close relatedness between *Thelandros* and *Ozolaimus* (Figs 2A, 3A and 3B).

The allocation of *P*. *sceleratus* in *Thelandros* has been focus of discussion [5,16], which leaded some authors to misallocate the species (e.g. [9]). The present results supported the validity of *P*. *sceleratus* instead of *T*. *sceleratus* as asserted by [16]. The species clusters within *Parapharyngodon* forming a well-supported assemblage (Fig 2A) and further analysis using nuclear and mitochondrial genetic markers would give more support to this conclusion.

The genera *Spauligodon* and *Skrjabinodon* appear as independent, closely related lineages. However, special attention should be given to the sequences KX550022 and KX550043. In one hand, these sequences were considered isolates of *Spauligodon* sp., named as ‘*Spauligodon* sp. type trimorphi’ in GenBank. On the other hand, [9] consider *Skrjabinodon trimorphi* as the isolation source of these same sequences. Unfortunately, the correct identification of this isolate could not be achieved because its morphological aspects were not fully represented (see S2 Fig in [9]). Consequently, we could not include *Spauligodon* sp. in the combined evidence tree due to this lack of morphological details. Tentatively, *Spauligodon* differs from *Skrjabinodon* based on the presence of caudal alae in males, but this trait appears to be homoplastic (Table 3) and it randomly occurs among other genera of Pharyngodonidae (see [1]), indicating that some systematic aspects within the family should be reviewed.

A curious situation was noted in the assemblage formed by *Spauligodon* spp. generated from concatenated gene sequences (Fig 3A). All the congeners clustered forming two well supported assemblages, with exception of *Spauligodon* sp. One assemblage included *S*. *saxicolae*, *S*. *lacerate*, *S*. *carbonelli* and *S*. *atlanticus*, parasites of skinks and the other included *S*. *nicolauensis* and *S*. *auziensis* parasites of geckos. These assemblages were formed independently from the geographic distribution (see Table 1 and Fig 3A for details), but accordingly to host taxa (i.e. Gekkonidae and Scincidae). This finding illustrate the early assertion of [44], posteriorly extrapolated by [2], in which lineages of pharyngodonid parasites in reptiles have been passing through a speciation process leaded by host capture. However, even though the present results agree with [2,44], much needs to be done before definitive conclusions.

The general phylogenetic pattern of the combined evidence tree (morphological + history traits + molecular data) (Fig 3A) agreed with that from the genetic analysis (Figs 2A and 2B, 3A). In this integrated approach, only samples identified to specific level were considered in order to keep the maximum morphological accuracy. Results related to the mapping of some morphological and life history traits should be interpreted with attention, because it may be biased for the nature of the dataset. An example is that the tail filament in males appears as non homoplastic, in which solely *Ozolaimus* showed the absence of this character. Even though tail filament seemingly holds phylogenetic information, no further conclusions can be achieved because other genera of Pharyngodonidae, e.g., *Alaeuris* and *Ortleppnema* show a reduced state of this character and still have not been genetically characterized. In the same context, it should be mentioned that the supposed homoplastic trait “host class” was purposely chosen to emphasize the ingroup composed only by parasites of reptiles, but we highlight that Pharyngodonidae allocates parasites from other host classes.

According to [2,45], two monophyletic lineages are recognized within pharyngodonid parasites of in reptiles. This distinction has been based upon the host dietary habits, vulval position in females, caudal structures in males and egg morphology. The present cladograms showed patterns formed according to these lineages: one included *Ozolaimus*, *Parapharyngodon* and *Thelandros*, and other included *Skrjabinodon* and *Spauligodon* (Figs 2A, 2B, 3A and 3B). However, the present results indicated that the morphological and life history traits do not reflect the phylogeny of these parasites. Host dietary habit shows considerable degree of homoplasy (Table 3) and was observed independently in several lineages of Pharyngodonidae (see Table 2 for details). These findings probably reflect the lack of knowledge on host biology at the time that [2,45] were published.

Vulval position was a non homoplastic trait (Table 3) and partially proper with to the assertion of [2,45]. The vulval location at the anterior region of body appeared as a synapomorphy of the assemblage formed by *Skrjabinodon* and *Spauligodon* (Fig 3B). The median vulval location was shared by *Parapharyngodon* and *Thelandros*; in *Ozolaimus*, sister group of *Thelandros*, the vulva near the posterior end of body appeared as a synapomorphy (Fig 3B). These observations suggest that changes in the vuval position seem to be consistent with the phylogeny of pharyngodonid nematodes parasitic in reptiles.

Several traits on the caudal region of males have been used for diagnosing the genera of Pharyngodonidae, e.g., presence of caudal alae, if the papillae are reduced, large or pedunculate, presence/absence of genital cone (see [4,45] for additional details). These traits could not be mapped as synapomorphic changes in the combined evidence tree (Fig 3B) and were highly homoplastic according to the results (Table 3). Therefore, it is hard to conclude if these character states represent cases of convergent evolution or if they were ancestral traits that have been lost in multiple occasions [10].

The combined evidence tree revealed an apparent inconsistence within the assemblage of *Spauligodon*. All representatives, except *S*. *nicolauensis* (see [11]), shared the following traits: lateral alae present in both male and female, and egg with two terminal opercula (Fig 3B). This caused a slight change on the topology of the combined evidence tree compared with that of concatenated genetic sequences (Fig 3A and 3B). It is plausible that [11] misinterpreted these characters, since they are easy to overlook; especially the eggs when not dissected from uterus will not show enough details. Furthermore, *S*. *nicolauensis* has been not studied using SEM.

The presence of lateral alae only in males has been considered in the differential diagnosis of *Thelandros* and *Parapharyngdon* [5]. However, according to [4] the most important features are related to the morphology of tail and eggs. Results from the combined datasets indicated weakness of tail structures on the generic diagnosis, whereas the egg morphology appears to be a strong character (Table 3, Fig 3B). In this sense,[46] and recently [47], considered the structure of eggs important for the taxonomy of Oxyurida. Thus, both *Thelandros* and *Parapharyngodon* should be considered valid and their differential diagnosis should be based on the eggs, until new data is available. Eggs of *Thelandros* have a polar terminal operculum and in those of *Parapharyngodon* the operculum is subterminal.

In addition to other features, [2] used the shape of eggs to separate the supposedly monophyletic lineages within Pharygodonidae into two subfamilies, i.e., Pharyngodoninae and Thelandroinae. The combined evidence tree and the CI for the character “egg shape” corroborate with this assertion (Fig 3B, Table 3). However, partitioning Pharyngodonidae in subfamilies should not be adopted, since genetic characterization of genera is still poor and the presence/absence of caudal alae in males (considered by [2] as an important trait) seems to be homoplastic and random (Table 3).

The present results may clarify some aspects on the relationship among genera of Pharyngodonidae parasitizing reptiles, as well as the importance of some morphological traits used on their diagnosis. However, this preliminary approach needs to be expanded in the future as the database improves; new genetic markers used and new genera included. The following conclusions could be achieved: (i) addition of new morphological and molecular data for *P*. *bainae* confirmed its validity, (ii) the closely-related genera *Skarjabinodon* and *Spauligodon* apparently are monophyletic and the presence/absence of caudal alae in males is the most evident difference between them, (iii) the relative position of vulva and the morphology of eggs seem to retain important phylogenetic information confirming the assertion of [2,45], (iv) characters of male caudal structure are highly homoplastic and their state variability could represent cases of convergent evolution or ancestral traits lost multiple times, (v) *Thelandros* and *Parapharyngodon* should be considered valid and their morphological distinction should be based exclusively on the egg structure as highlighted by [8].

## Supporting information

S1 Fig Most parsimonious tree from mmorphological-life history traits matrix of pharingodonid nematodes parasitic in reptiles, generated from Heuristic Search in PAUP. (PDF)

S2 Fig Maximum likelihood (ML) tree of the sequences of 18S rDNA from pharyngodonid nematodes parasitic in reptiles, showing bootstrap values (1,000 replications). (PDF)

S3 Fig Maximum likelihood (ML) tree of the sequences of 28S rDNA from pharyngodonid nematodes parasitic in reptiles, showing bootstrap values (1,000 replications). (PDF)

S4 Fig Maximum likelihood (ML) tree of the concatenated sequences of 18S and 28S rDNA from pharyngodonid nematodes parasitic in reptiles, showing bootstrap values (1,000 replications). (PDF)

S1 Table Morphometry (in range) of *Parapharyngodon bainae* Pereira, Sousa & Souza-Lima, 2011 parasite of *Tropidurus torquatus* (Wied-Neuwied, 1820) from Toledos, Juiz de Fora, State of Minas Gerais, Brazil, collected in the present study. All measurements are given in micrometers unless otherwise stated.
(PDF)
