# Supplementary material for: Integrative approach on Pharyngodonidae (Nematoda: Oxyuroidea) parasitic in reptiles: Relationship among its genera, importance of their diagnostic features, and new data on Parapharyngodon bainae
Source: PLoS One. 2018 Jul 11;13(7):e0200494. doi: 10.1371/journal.pone.0200494 (PMC6040771; doi:10.1371/journal.pone.0200494)
Supplement: S1 Fig — (PDF) [file pone.0200494.s001.pdf]

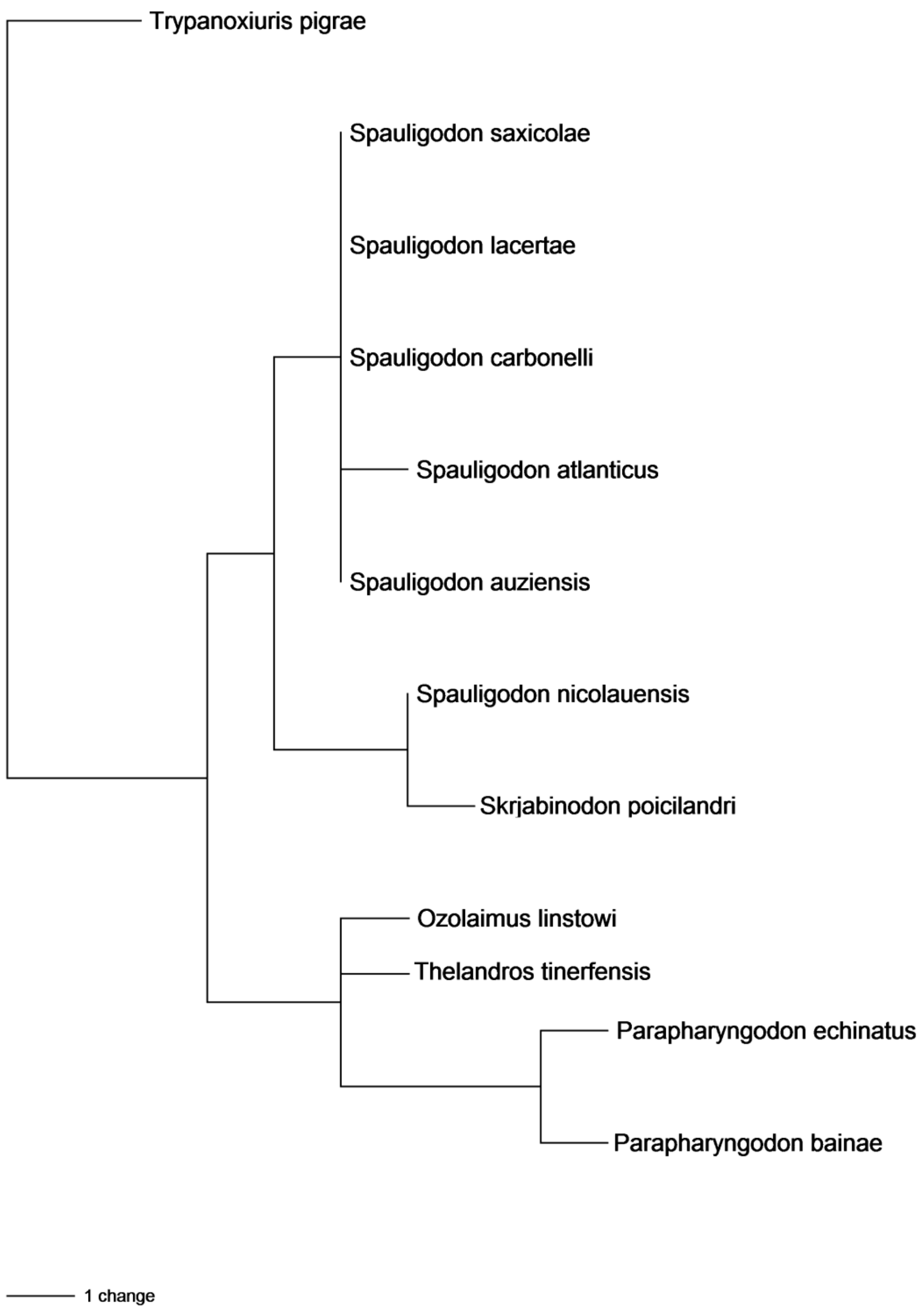

S1 Fig. Most parsimonious tree from morphological-life history traits matrix of pharyngodonid nematodes parasitic in reptiles, generated from Heuristic Search in PAUP.
