# Supplementary material for: Integrative approach on Pharyngodonidae (Nematoda: Oxyuroidea) parasitic in reptiles: Relationship among its genera, importance of their diagnostic features, and new data on Parapharyngodon bainae
Source: PLoS One. 2018 Jul 11;13(7):e0200494. doi: 10.1371/journal.pone.0200494 (PMC6040771; doi:10.1371/journal.pone.0200494)
Supplement: S2 Fig — (PDF) [file pone.0200494.s002.pdf]

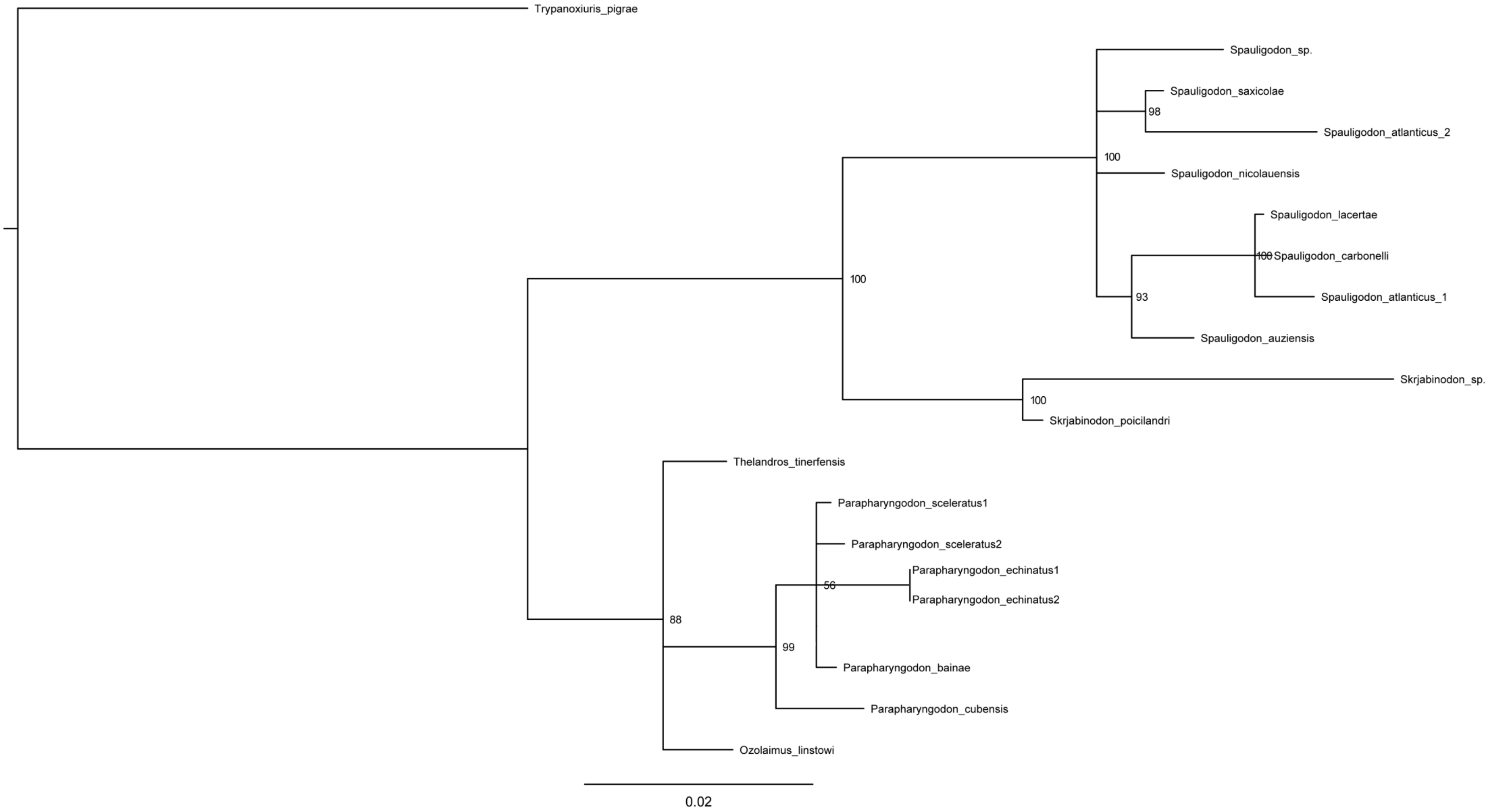

S2 Fig. Maximum likelihood (ML) tree of the sequences of 18S rDNA from pharyngodonid nematodes parasitic in reptiles, showing bootstrap nodal supports (1,000 replications).
