# Supplementary material for: Integrative approach on Pharyngodonidae (Nematoda: Oxyuroidea) parasitic in reptiles: Relationship among its genera, importance of their diagnostic features, and new data on Parapharyngodon bainae
Source: PLoS One. 2018 Jul 11;13(7):e0200494. doi: 10.1371/journal.pone.0200494 (PMC6040771; doi:10.1371/journal.pone.0200494)
Supplement: S1 Table — All measurements are given in micrometers unless otherwise stated. (PDF) [file pone.0200494.s005.pdf]

S1 Table. Morphometry (in range) of *Parapharyngodon bainae* Pereira, Sousa & Souza Lima, 2011 parasite of *Tropidurus torquatus* (Wied-Neuwied, 1820) from Toledos, Juiz de Fora, State of Minas Gerais, Brazil, collected in the present study. All measurements are in micrometers unless otherwise stated.

	**Males (n = 15)**	**Females (n = 15)**
**Body length (mm)**	3.3–3.6	6.0–7.5
**Maximum width**	250–350	800–900
**Nerve ring to anterior end**	120–142	200–233
**Excretory pore to anterior end (mm)**	1.2–1.3	1.7–1.9
**Total length of oesophagus**	500–550	1,100–1,300
**Oesophageal bulb length**	100–130	180–200
**Oesophageal bulb width**	100–140	220–266
**Spicule length**	99–135	-
**Tail filament length**	90–100	-
**Tail length**	-	500–610
**Tail spike**	-	200–225
**Vulva to anterior end (mm)**	-	3.0 – 3.6
**Egg length × width**	-	85–95 × 55–63
